# Supplementary figures and images for: Suppressive impact of metronomic chemotherapy using UFT and/or cyclophosphamide on mediators of breast cancer dissemination and invasion
Source: PLoS One. 2019 Sep 19;14(9):e0222580. doi: 10.1371/journal.pone.0222580 (PMC6752870; doi:10.1371/journal.pone.0222580)

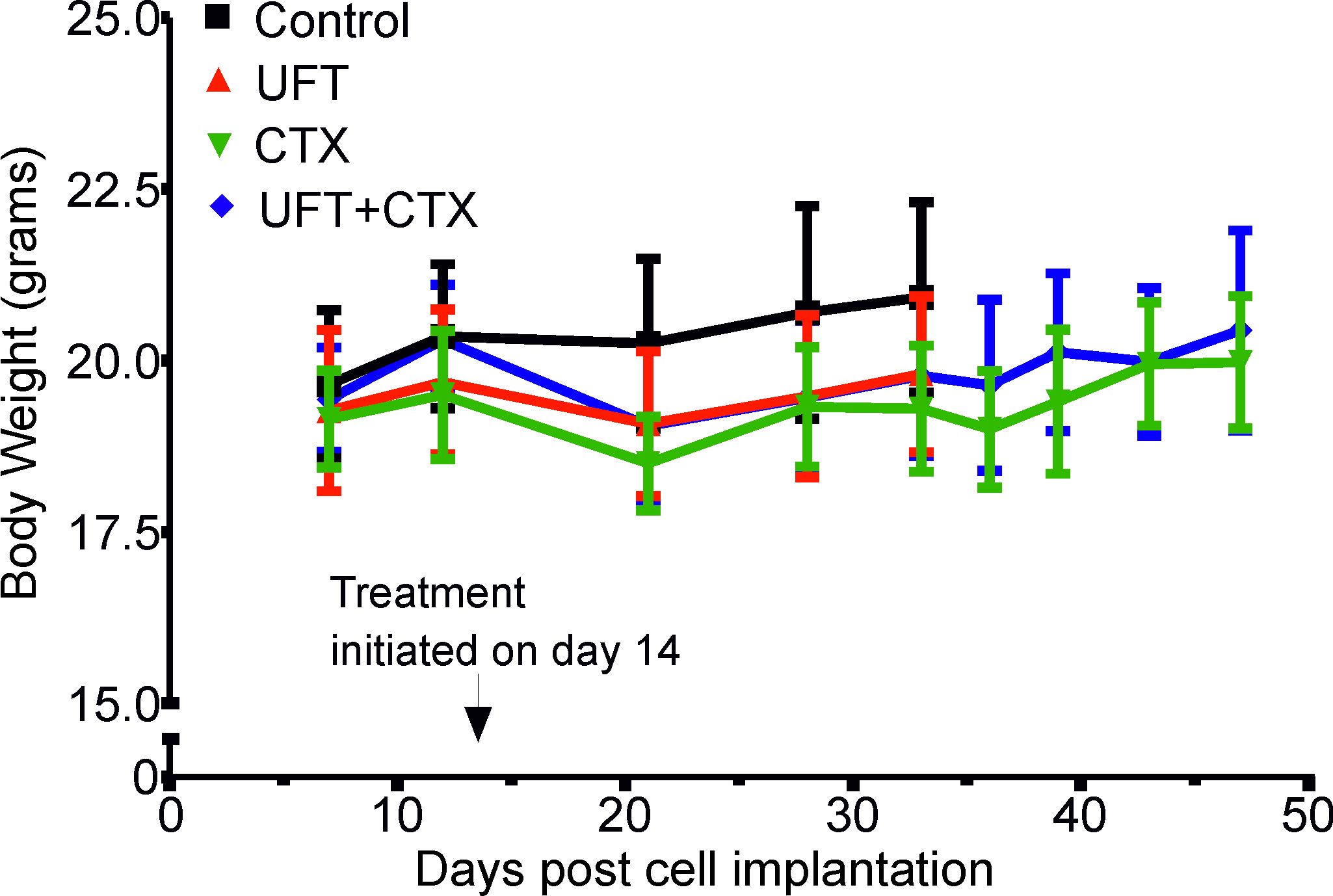

Supplement: S1 Fig — (TIF) [file pone.0222580.s001.tif]

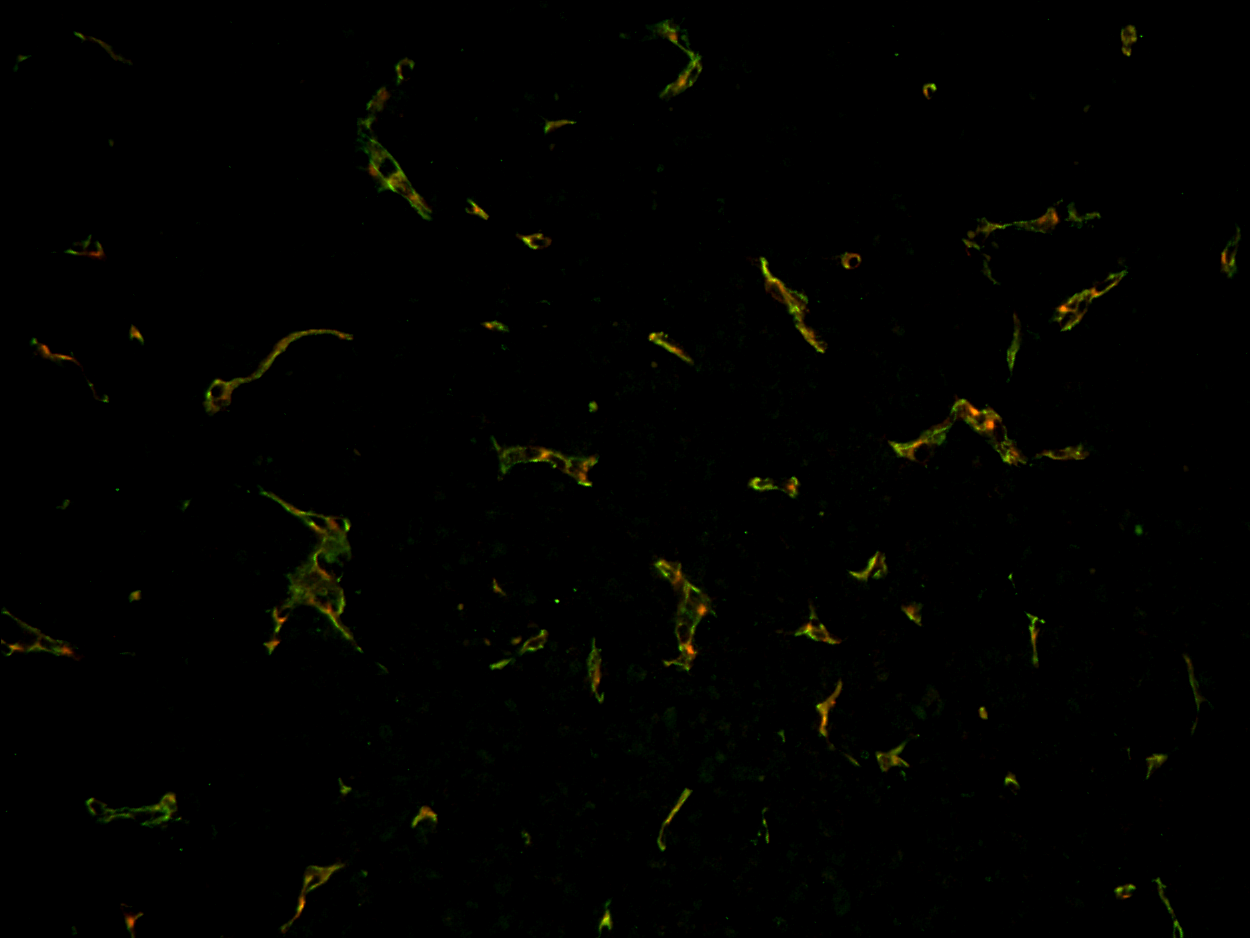

Supplement: S2 Fig — Dual immunofluorescence staining for CD31 (green) and VEGFR2 (red) in 231/LM2-4 tumor samples. (TIF) [file pone.0222580.s002.tif]

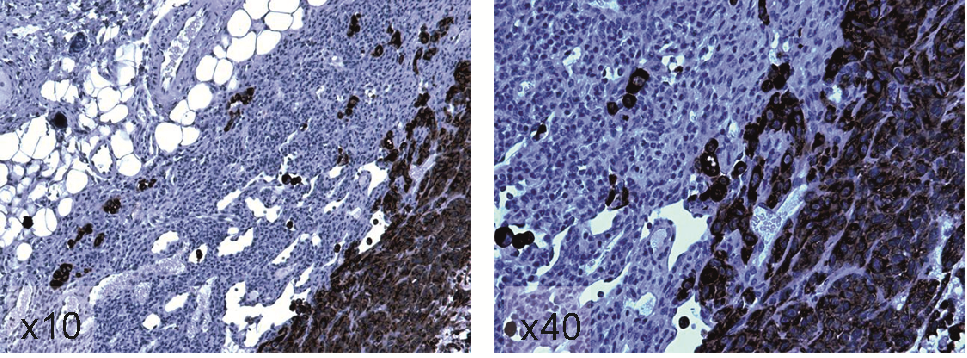

Supplement: S3 Fig — Vimentin staining shows the invasive front of the tumor and the tumor cells invading the stroma. The primary antibody is specific to human vimentin. The negative control of the immunostaining is therefore the mouse tissue itself. (TIF) [file pone.0222580.s003.tif]

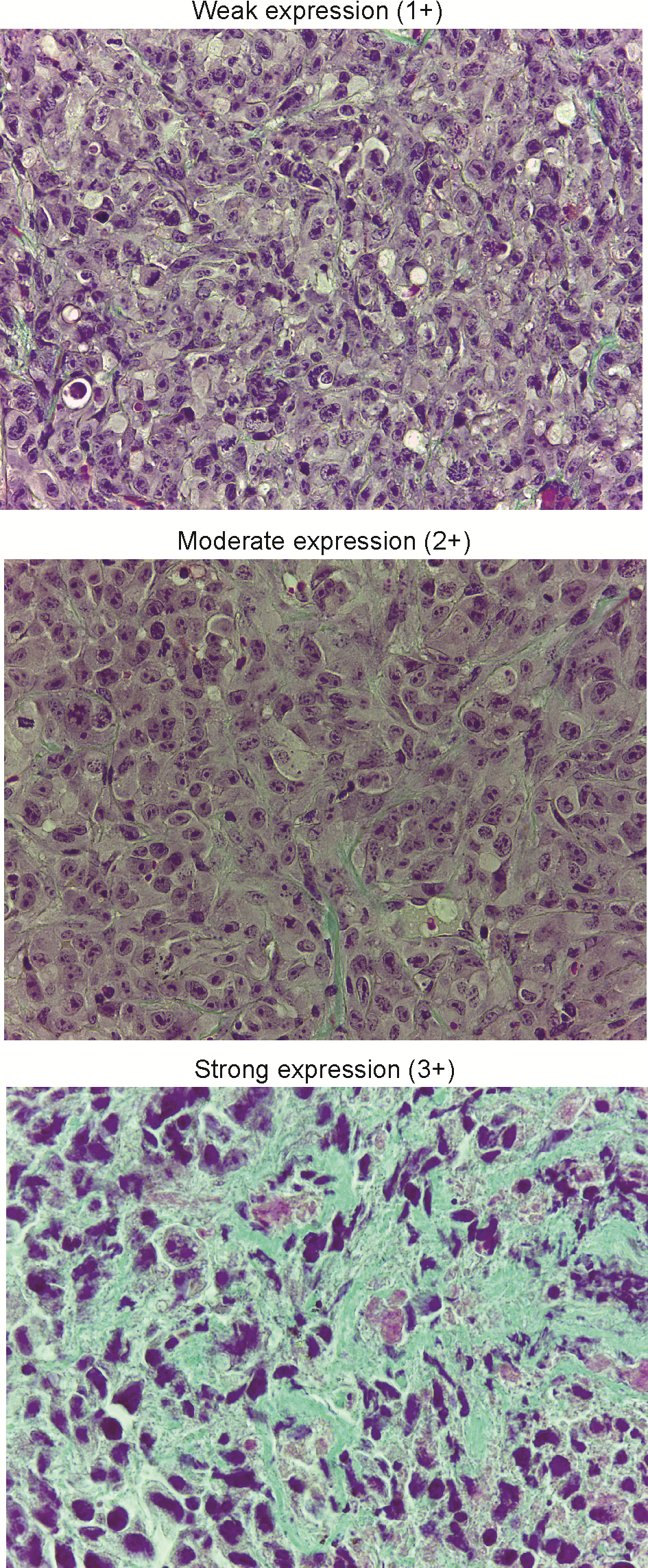

Supplement: S4 Fig — We used a grading system composed of mild/focal (grade 1), moderated (grade 2), and extensive (grade 3) collagen deposition. (TIF) [file pone.0222580.s004.tif]

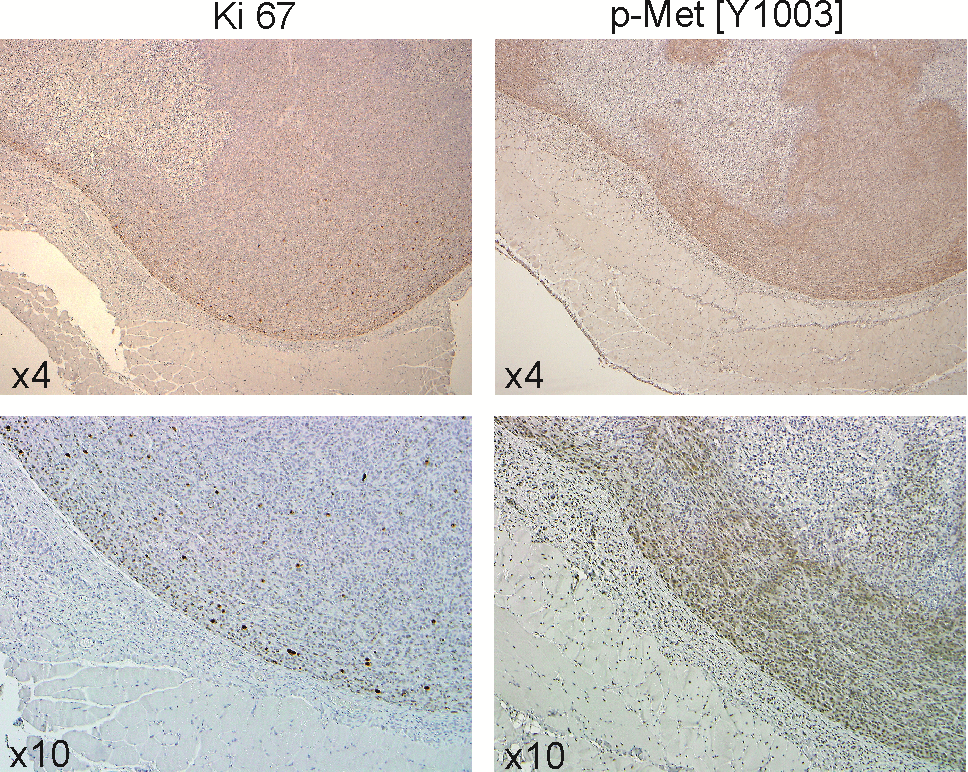

Supplement: S5 Fig — Areas of positive staining overlap. (TIF) [file pone.0222580.s005.tif]

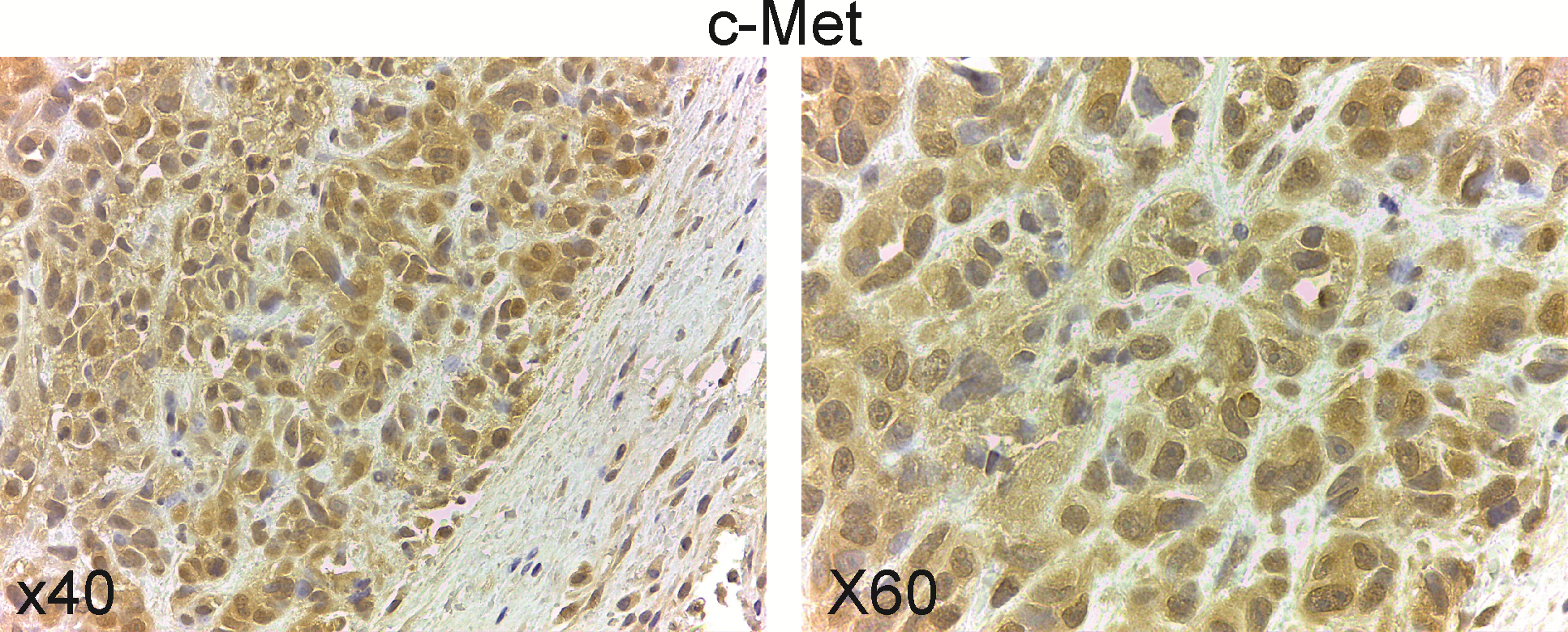

Supplement: S6 Fig — A strong nuclear and cytoplasmic expression of c-Met was observed in all treatment groups, with no noticeable difference. (TIF) [file pone.0222580.s006.tif]

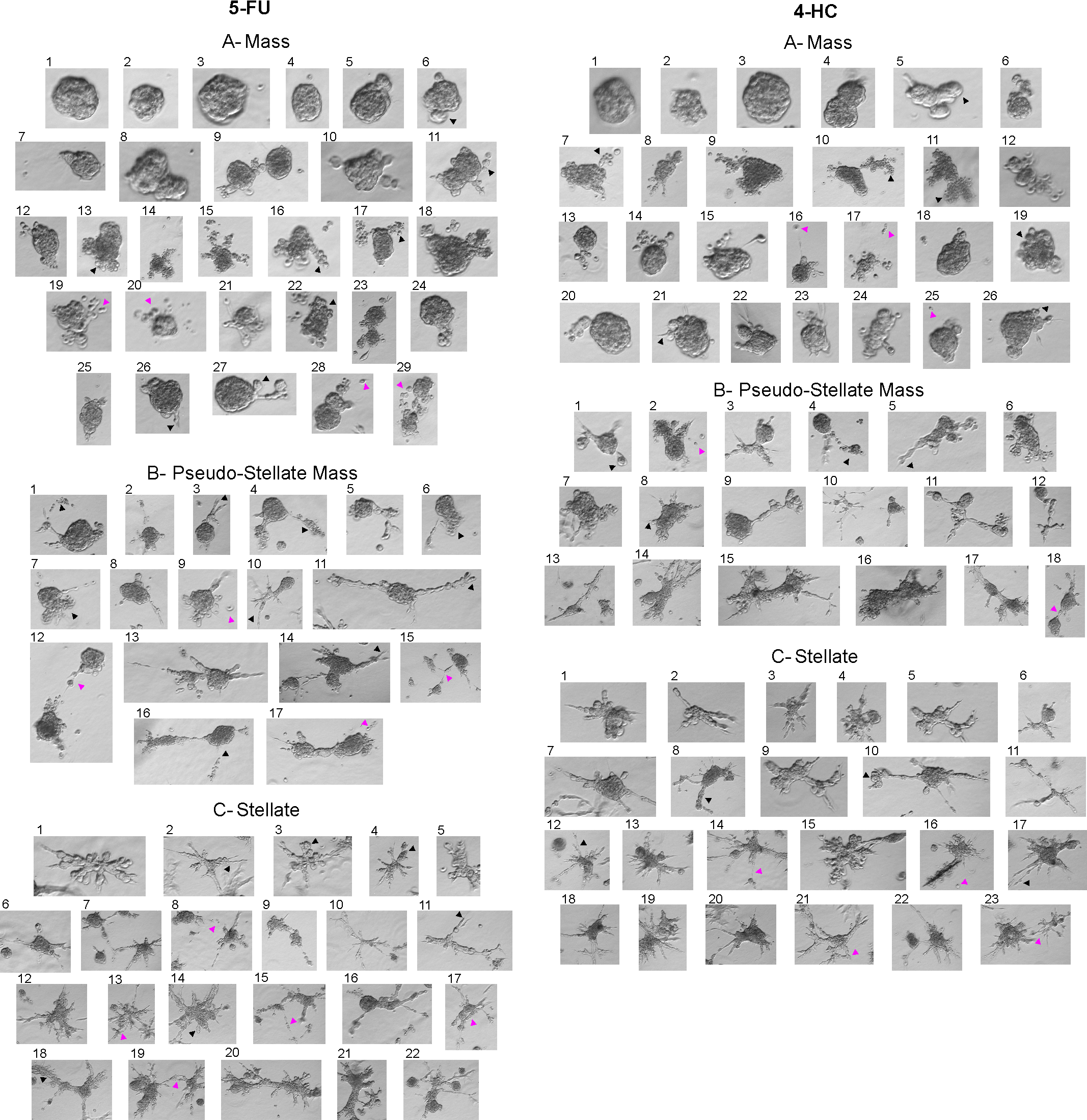

Supplement: S7 Fig — Representative examples of the different morphological phenotypes of the multicellular structures. (1μM 5-FU): Mass structures (A): round morphology (1–4), collective cell migration as chains of few cells with smooth borders (Δ11,16), buds (Δ6), or as disorganized masses (Δ22). Single-cell protrusions (Δ26,27). Multicellular streaming with no apparent junction contacts (Δ13,17). Dissemination of single tumor cells (pink Δ20,28) and group of cells (pink Δ19,29). Pseudo-Stellate Mass structures (B): multicellular collective protrusive migration with leading cells with invadopodia (Δ3,10,14) or leading buds (Δ11), and a loose assembly of individual round cells in multicellular structures (Δ1,4,6,7). Dissemination of single tumor cells (pink Δ9) and group of cells (pink Δ17). Contact (pink Δ12) and fusion (pink Δ15) between different structures. Stellate structures (C): protrusive leading front with invadopodia (Δ11) or leading buds (Δ3,4,14). Multicellular invasive chains with 1–2 cells in diameter (Δ2) or broad masses of cells (Δ18). Collective cell dissemination (pink Δ15). An uncoordinated arrangement of the component cells in some multicellular chains (pink Δ13,17), contacts (pink Δ8,19), fusions (images 16,20,22) between different structures to form a large stellate structure. (0.01μM 4-HC): Mass structures (A): round morphology (1–3), collective cell migration as chains of few cells with smooth borders (Δ7), buds (Δ5), or as disorganized masses (Δ19). Single-cell protrusions (Δ21,26). Multicellular streaming with no apparent junction contacts (Δ10,11). Dissemination of single tumor cells (pink Δ16,25) and group of cells (pink Δ17). Pseudo-Stellate Mass structures (B): multicellular collective protrusive migration pattern containing leading cells with invadopodia (Δ5,8) or leading buds (Δ1), and a loose assembly of individual round cells in multicellular structures (Δ4). Dissemination of single tumor cells (pink Δ2). Fusion between different structures (pink Δ18). [file pone.0222580.s007.tif]
