# Supplementary material for: Suppressive impact of metronomic chemotherapy using UFT and/or cyclophosphamide on mediators of breast cancer dissemination and invasion
Source: PLoS One. 2019 Sep 19;14(9):e0222580. doi: 10.1371/journal.pone.0222580 (PMC6752870; doi:10.1371/journal.pone.0222580)
Supplement: S1 Table — (DOCX) [file pone.0222580.s008.docx]

**S1 Table.** **Assessment of peritumoral and intratumoral collagen deposition in paraffin tumor sections**.

| **Slide number** | **Collagen peritumor** | **Collagen intratumor** |
| --- | --- | --- |
| Control |  |  |
| T1 | 2-3 | 3 |
| T2 | 2 | 2-3 |
| T3 | 2 | 2-3 |
| T4 | 1 | 3 |
| T5 | 0-1 | 2-3 |
| UFT |  |  |
| T1 | 1 | 2-3 |
| T2 | 1 | 1-2 |
| T3 | 2 | 2-3 |
| T4 | 2 | 2 |
| T5 | 2 | 1-2 |
| CTX |  |  |
| T1 | 2 | 2 |
| T2 | 2 | 2 |
| T3 | 3 | 1-2 |
| T4 | 1 | 2 |
| UFT + CTX |  |  |
| T1 | 2 | 3 |
| T2 | 1 | 1 |
| T3 | 1 | 2 |
| T4 | 1 | 2 |
| T5 | 0-1 | 3 |

Masson´s trichrome histochemical staining was analysed by a grading system composed of mild/focal (grade 1), moderated (grade 2) and extensive (grade 3) collagen deposition. The terms T1, T2, etc., refer to the number of the tumor, each of which was resected from a different mouse in the specified treatment group.
