# Supplementary material for: Suppressive impact of metronomic chemotherapy using UFT and/or cyclophosphamide on mediators of breast cancer dissemination and invasion
Source: PLoS One. 2019 Sep 19;14(9):e0222580. doi: 10.1371/journal.pone.0222580 (PMC6752870; doi:10.1371/journal.pone.0222580)
Supplement: S2 Table — (DOCX) [file pone.0222580.s009.docx]

**S2 Table. Assessment of p-Met[Y1003] in paraffin tumor sections**.

| **Slide number** | **p-Met[Y1003] score** |
| --- | --- |
| Control |  |
| T1 | 3 |
| T2 | 2 |
| T3 | 3 |
| T4 | 2 |
| T5 | 1 |
| UFT |  |
| T1 | 2 |
| T2 | 2 |
| T3 | 1 |
| T4 | 2 |
| T5 | 3 |
| CTX |  |
| T1 | 1 |
| T2 | 2 |
| T3 | 2 |
| T4 | 2 |
| UFT + CTX |  |
| T1 | 1 |
| T2 | 1 |
| T3 | 1 |
| T4 | 1 |
| T5 | 2 |

The intensity of p-Met[Y1003] staining was evaluated using the following grading system: weak (grade 1), moderated (grade 2) and strong (grade 3). The terms T1, T2, etc., refer to the number of the tumor, each of which was resected from a different mouse in the specified treatment group.
