# Supplementary material for: Suppressive impact of metronomic chemotherapy using UFT and/or cyclophosphamide on mediators of breast cancer dissemination and invasion
Source: PLoS One. 2019 Sep 19;14(9):e0222580. doi: 10.1371/journal.pone.0222580 (PMC6752870; doi:10.1371/journal.pone.0222580)
Supplement: S1 Appendix — (DOCX) [file pone.0222580.s010.docx]

**S1 Appendix. Assessment of the anti-metastatic effect associated with UFT+CTX therapy in the neoadjuvant setting in 231/LM2-4 breast cancer model.** 2x10^6^ 231/LM2-4 cells (highly metastatic variant of the human MDA-MB-231) [1] were orthotopically implanted in the right inguinal mammary fat pads [1] of female 6-8 week-old yellow fluorescent protein (YPF) SCID mice [2]. Weekly caliper measurement were performed to determine tumor growth and tumor volume (mm^3^) was calculated using the formula a^2^ x b/2 where a is the width and b the length. All mice were randomized just before initiation of treatment in four groups (n=9-10 per group). Control treatment group: 0.1% HPMC per oral (p.o.) once daily; UFT treatment group: 15 mg/Kg/d UFT p.o. once daily; CTX treatment group 20 mg/Kg/d CTX through the drinking water and 0.1% HPMC p.o. daily and the combination UFT + CTX treatment group: 15 mg/Kg/d UFT in combination with 20 mg/Kg/d CTX through the drinking water. The treatment was initiated, on day 14th after cell implantation, when average tumor volume was approximately 200 mm^3^ and it was maintained during nine days. Then, primary tumors were resected. All mice were sacrificed 14 days after primary tumor resection. The extent of metastatic disease in bone and lung was assessed by means of vimentin immunohistochemistry staining. The levels of vimentin staining were then examined using pixel quantification with Adobe Photoshop and expressed as arbitrary units (AU) .The results of metastatic disease in bone are shown in the Figure. One-Way ANOVA with Tukey’s multiple comparison shows that UFT+CTX is significantly lower (P<0.05) than either control or CTX groups.


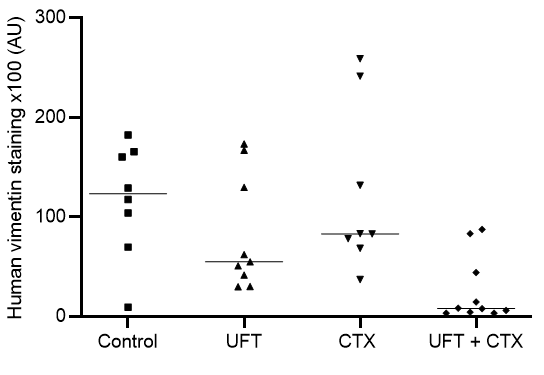


**References:**

1. Munoz R, Man S, Shaked Y, Lee C, Wong J, Francia G, et al. Highly efficacious non-toxic treatment for advanced metastatic breast cancer using combination UFT-cyclophosphamide metronomic chemotherapy. Cancer Res. 2006;66:3386-3391.

2. Tait LR, Pauley RJ, Santner SJ, Heppner GH, Heng HH, Rak JW et al. Dynamic stromal-epithelial interactions during progression of MCF10DCIS.com xenografts. Int J Cancer. 2007;120:2127–34.
