# Supplementary material for: Suppressive impact of metronomic chemotherapy using UFT and/or cyclophosphamide on mediators of breast cancer dissemination and invasion
Source: PLoS One. 2019 Sep 19;14(9):e0222580. doi: 10.1371/journal.pone.0222580 (PMC6752870; doi:10.1371/journal.pone.0222580)
Supplement: Attachment — Submitted filename: renamed_d5c34.docx [file pone.0222580.s011.docx]

**Responses to each point raised by the academic editor:**

2. To comply with PLOS ONE submissions requirements, in your Methods section, please provide additional information regarding the experiments involving animals and ensure you have included details on (1) methods of sacrifice at the end of the study, (2) methods of anesthesia and/or analgesia, and (3) efforts to alleviate suffering.

(1) Methods of sacrifice at the end of the study: the information is added in the section “*In vivo* tumor growth assessment” of Materials and methods; lines 663-666.

(2) Methods of anesthesia and/or analgesia: the information is added in the section “*In vivo* tumor growth assessment” of Materials and methods; lines 656-659.

(3) Efforts to alleviate suffering: the information is added in the section “*In vivo* tumor growth assessment” of Materials and methods; lines 656-659, 667-670.

3. We note that you have included the phrase "data not shown" in your manuscript. Unfortunately, this does not meet our data sharing requirements. PLOS does not permit references to inaccessible data. We require that authors provide all relevant data within the paper, Supporting Information files, or in an acceptable, public repository. Please add a citation to support this phrase or upload the data that corresponds with these findings to a stable repository (such as Figshare or Dryad) and provide and URLs, DOIs, or accession numbers that may be used to access these data. Or, if the data are not a core part of the research being presented in your study, we ask that you remove the phrase that refers to these data.

(1) No weight loss or other signs of toxicity were observed in any of the treatment groups (data not shown) lines 193-194. These data are now presented in supporting information (S1 Fig).

(2) Also observed was a small trend of increased intratumoral collagen deposition in the tumors that are more aggressive and show less extratumoral capsule formation (data not shown) lines 302-304. This information has been eliminated from the manuscript for being an observation and not being supported by a statistical analysis.

**Responses to each point raised by reviewer # 1:**

1. The title is unclear, please rephrase it. The title has been changed to “Suppressive impact of metronomic chemotherapy using UFT and/or cyclophosphamide on mediators of breast cancer dissemination and invasion”.

2. The introduction, even if it is clear and well written, is too long. It must be shortened. The Introduction section has been shortened. The information that has been removed is marked (yellow/dashed) in the “Revised Manuscript with Track Changes”.

3. In the results sections, Munoz and colleagues also include a discussion about the data. The authors should comment the data specifically in the discussion part. Furthermore, the titles of the paragraphs in the results section must be explicative of the data presented. Do not use general terms as "effects" and "assessment", please focus more on the take-on message.

(1) The authors should comment the data specifically in the discussion part. All paragraphs in the Results section, that included a discussion of the data have been removed and are now included in the Discussion section. The paragraphs eliminated and added have been marked (grey/dashed and grey respectively) in the corresponding sections of the “Revised Manuscript with Track Changes”.

(2) The titles of the paragraphs in the results section must be explicative of the data presented. The titles of the different subsections of the Results section have been modified in the manuscript to address this issue and correspond to the lines 147, 181, 218, 267, 290 and 365.

4. In general, I suggest to the authors to present the data reported in the tables with graphs. The raw data could be uploaded as supplementary materials. Also please, provide statistical analysis and a legend for the data in table 1.

(1) I suggest to the authors to present the data reported in the tables with graphs. The data of Table 1 is now presented in the Fig 3, which gives a clearer perspective of overall invasion. As suggested, the data of Table 2 is now presented in the Fig 4. The raw data have been uploaded as supporting information (S1 Table).

(2) Provide statistical analysis and a legend for the data in table 1. The statistical analysis of the data from Table 1, corresponding to the new Fig 3 in the manuscript is presented in lines 245-248 and in lines 262-265 of Fig 3 legend.

5. The main scope of this manuscript is to shed light on the mechanism beyond the observation that UFT, either alone or in combination with CTX, had a modest effect on primary tumor growth, but is able to inhibit metastatic spread. My main concern regards the fact that the authors based most of the evidence from a previous paper (Munoz R et al, 2006). Have the authors performed new experiments to evaluate lung metastasis at mice sacrifice or after tumor resection? If not, they should provide them to corroborate the previous evidences. Additional study has been conducted in the adjuvant setting and showing enhanced metastatic disease in bone. Relative information have been added in Discussion section (lines 603-611) and supporting information have been uploaded (S1 Appendix) that might address some of the concerns presented by the reviewer.

**Responses to each point raised by reviewer # 2:**

1. Why did the authors not confirm their 2006 finding of inhibition of micrometastases by the UFT + CTX combination? Tumor cell models can drift/evolve over time (13 years, in this case), and there is some concern that the in vivo findings with primary tumors, reported here, might not be directly applicable to the earlier findings of inhibition of metastasis in what may only nominally be the same tumor model. Further, if the prior metastasis study was performed in a post-surgical model, then the current findings about impact of UFT + CTX on metastatic potential in a non-surgical model may not be directly relevant. Additional study has been conducted in the adjuvant setting and showing enhanced metastatic disease in bone. Relative information have been added in Discussion section (lines 603-611) and supporting information have been uploaded (S1 Appendix).

2. In several places, conclusions stated in the text need to be based on a more rigorous implementation of statistical analysis. For example:

- Line 177 - Fig. 1 conclusion of delayed tumor growth needs statistical support. (Also, mark day of first drug treatment along the x-axis). Statistical support relative to Fig 1 is added (lines 154-156). The start of treatment has been marked on the Fig 1.

- Line 281 – Any conclusion of reduced invasion into adjacent soft tissue requires statistical analysis of Table 1 results. The data of Table 1 is now presented in the Fig 3 as suggested by reviewer #1.The statistical analysis of the data is presented in lines 245-248 and in lines 261-265 of Fig 3 legend.

- Line 300 – Similarly, the conclusion stated in the text of reduced collagen deposition in all treatment groups requires statistical analysis of Table 2 data. Text on line 302 indicates that is not significant. The data of Table 2 is now presented in the Fig 4 and the raw data have been uploaded as supporting information (S1 Table) as suggested by reviewer #1.The statistical analysis of the data is presented in lines 277-278 and in lines 286-288 of Fig 4 legend.

- Line 387 – Staining scores should be listed for each individual tumor in a supplemental Table, similar to the format of Table 2. The p-Met[Y1003] staining scores are listed in supporting information (S2 Table).

3. The entire work is apparently based on a single in vivo experiment (Fig. 1) with only 4-5 tumors per group. This limitation should be explicitly noted, e.g., in Discussion. This limitation has been noted in lines 602-603 of the Discussion section.

4. What is the impact of drug treatment on MMP expression – in vitro or in vivo (line 464)? The impact of the drug treatment on MMP expression was not extensively examined but this it is the focus of ongoing investigations. Due to the absence of definitive evidence of alterations in MMPs expression and activity by the treatments, the section related to MMPs has been removed.

5. Is the in vitro HC concentration really 10nM? Are there any prior studies showing a biological effect of HC at such a low concentration? That concentration is 100-500-fold lower that typically reported anti-tumor activities for HC. The cellular viability assays showed in Fig 9A show the effect of a range of concentrations of 5-FU (0.1, 0.5, 1 and 10μM) and of 4-HC (0.01, 0.05, 0.1 and 1μM). This assay provided us the concentrations 1μM 5-FU and 0.01μM 4-HC used for drug combination. Continuous treatment of the cells over six days with the aforementioned drugs concentrations had little effect on cell viability.

In all *in vitro* assays, we followed the experimental design from Bocci G et al (reference 75) that is a continuous low-dose exposure to the drugs for 6 days; every day the medium was removed and fresh drugs were added to the cells with new medium.

In the article: Zanotto-Filho A et al. Cancer Lett. 2018;425:101–115, a viability assay in MDA-MB231 cells with 4-HC at 25μM (indicated as IC_50_ value) for 48h of treatment was done. In our viability assay in 231/LM2-4 with 4-HC for 6 days of treatment the IC_50_ level was around 0.4μM.

6. Minor Points

- Shorten Introduction by editing.The Introduction section has been shortened. The information that has been removed is marked (yellow/dashed) in the “Revised Manuscript with Track Changes”.

- Line 61 – Correct text to indicate clinical trial metronomic (?) studies. The text in line 60 has been corrected to include the word “metronomic”.

- Line 381 – Arrow not visible on Figure. Fig 3 has been separated in two figures - Fig 5 and Fig 6 - in order to meet PLoS One requirements. The arrows in both Figures are visible.

- Table 3 – Specify drug concentration. Same for Figure S6, etc. (see line 631). Table 3 corresponds to Table 1 in the revised manuscript, the drug concentrations used have been specified in these tables and in the rest of the figures.

- Line 617 ff. Specify sources for HC. The source of 4-HC used in the study have been specified in line 641.
